# Motion State Estimation of Target Vehicle under Unknown Time-Varying Noises Based on Improved Square-Root Cubature Kalman Filter

**DOI:** 10.3390/s20092620

**Published:** 2020-05-04

**Authors:** Shiping Song, Jian Wu

**Affiliations:** State Key Laboratory of Automotive Simulation and Control, Jilin University, Changchun 130022, China; songsp17@mails.jlu.edu.cn

**Keywords:** millimeter-wave radar, square-root cubature Kalman filter, Sage-Husa algorithm, target tracking, stationary and moving object classification

## Abstract

In the advanced driver assistance system (ADAS), millimeter-wave radar is an important sensor to estimate the motion state of the target-vehicle. In this paper, the estimation of target-vehicle motion state includes two parts: the tracking of the target-vehicle and the identification of the target-vehicle motion state. In the unknown time-varying noise, non-linear target-vehicle tracking faces the problem of low precision. Based on the square-root cubature Kalman filter (SRCKF), the Sage–Husa noise statistic estimator and the fading memory exponential weighting method are combined to derive a time-varying noise statistic estimator for non-linear systems. A method of classifying the motion state of the target vehicle based on the time window is proposed by analyzing the transfer mechanism of the motion state of the target vehicle. The results of the vehicle test show that: (1) Compared with the Sage–Husa extended Kalman filtering (SH-EKF) and SRCKF algorithms, the maximum increase in filtering accuracy of longitudinal distance using the improved square-root cubature Kalman filter (ISRCKF) algorithm is 45.53% and 59.15%, respectively, and the maximum increase in filtering the accuracy of longitudinal speed using the ISRCKF algorithm is 23.53% and 29.09%, respectively. (2) The classification and recognition results of the target-vehicle motion state are consistent with the target-vehicle motion state.

## 1. Introduction

Millimeter-wave radar is an important sensor that constitutes an advanced driver assistance system (ADAS). The estimation of the moving state of the target vehicle based on the on-board millimeter-wave radar is essential for predicting the future trajectory of the target vehicle and determining the degree of danger of the target vehicle to the ego vehicle. In this paper, the motion state estimation of the target vehicle includes target-vehicle tracking and target-vehicle motion state classification. 

The motion state information of the target vehicle (relative radial distance, azimuth, and relative radial rate) measured by millimeter-wave radar is obtained from the polar coordinate system. However, in the process of target tracking, the target motion model is usually established in the Cartesian coordinate system. As can be seen from the radar target-tracking process, the state equation is linear and the measurement equation is non-linear. Since the measurement equation is non-linear, the target-tracking system based on the millimeter-wave radar is a non-linear system.

Extended Kalman filter (EKF) [1,2], unscented Kalman filter (UKF) [3,4], particle filter (PF) [5] and cubature Kalman filter (CKF) [6] are common non-linear filtering state estimation algorithms. 

The basic idea of EKF is: the non-linear system is linearized by Taylor series expansion, and then Kalman filtering is performed. Inaccurate modeling of system noise and changes in model parameters due to environmental factors will cause the decrease of the EKF estimation accuracy, and even the filter divergence will be caused. To ensure the accuracy and stability of EKF under unknown and time-varying conditions, many scholars carry out research on the adaptive extended Kalman filter algorithm, adaptive extended Kalman filter (AEKF) algorithm, such as Sage–Husa’s maximum a posteriori estimation [7], fictitious noise compensating [8], dynamic bias decoupling estimation [9], etc. To solve the filtering divergence problem caused by system modeling errors, some scholars proposed an EKF with a suboptimal fading factor [10]. Meanwhile, to improve the estimation accuracy of the EKF algorithm, some scholars proposed an AEKF algorithm based on a neural network [11]. The adaptive learning characteristics of neural networks are used to identify the non-linear system model online, to overcome the influence of unmodeled dynamic characteristics of the filter. However, due to the following shortcomings of the EKF algorithm, its development in engineering practice is limited:

(1) The higher-order terms are truncated (with truncation error) and only the first-order terms retained during the Taylor series are expanded, and the accuracy of EKF is the first-order Taylor series;

(2) In many engineering applications, the Jacobian matrix of the measurement equation is difficult to solve.

The basic idea of UKF is: “It is easier to approximate the probability density distribution of non-linear functions than the approximation of non-linear functions” [12]. UKF uses the unscented transformation to approximate the posterior distribution of the state of the non-linear system. The most important part of the UKF algorithm is the sampling strategy. Different sampling strategies differ in the number, location, and corresponding weights of the extracted Sigma points [13]. Compared with EKF, UKF has the following two advantages:

(1) The accuracy of UKF can reach at least two orders under the condition of EKF, and UKF algorithm takes the same order of magnitude;

(2) In UKF, it is not necessary to calculate the Jacobian matrix of the measurement equation.

The above two advantages of UKF expand the application range of the EKF algorithm. However, certain sigma point weights ω < 0 will cause the covariance matrix to non-positive definite condition when the dimension is too high (N ≥ 4). This situation will lead to the following two effects: firstly, the filter value is not stable or even divergent; secondly, the dimension disaster problem will occur [14]. Therefore, some scholars through theoretical analysis and experiments have proved that UKF has high accuracy for low-dimensional (N ≤ 2) non-linear systems [15].

The basic idea of PF is to approximate the posterior probability density function of the system state by random particles. PF uses the particle mean value instead of the integral operation to obtain the minimum variance of state. With the increase in the number of particles, the probability density function of particles gradually approximates the probability density function of the real state. However, PF has the following two shortcomings, which restrict the development of PF [16]:

(1) the particle degradation problem;

(2) It is difficult to realize online estimation due to the large amount of computation.

To better solve the problem of poor filtering performance and even divergence in high-dimensional non-linear filtering estimation, Arasaratnam and Haykin proposed a third-degree spherical-radial rule CKF [17]. After CKF was proposed, it was widely used in target tracking [18] and navigation [19]. Compared with UKF, CKF has the following advantages:

(1) The UKF algorithm selects 2n+1 sampling points with different weights, while the CKF algorithm selects 2n sampling points with the same weight. The CKF algorithm has fewer sampling points than UKF, so the CKF algorithm takes less time than UKF.

(2) Since the weight coefficients of the sampling points of the CKF algorithm are all positive, the robustness of the CKF algorithm is high when the dimension of the observed variable is too high (N ≥ 4).

In conclusion, compared with EKF and UKF, CKF has higher estimation accuracy. During the operation of the standard CKF algorithm, the following two conditions should be met: (1) symmetry, (2) positive qualitative. However, in practical engineering, these two conditions are sometimes difficult to meet. Therefore, scholars proposed the SRCKF algorithm based on the CKF algorithm [20]. SRCKF has the following two advantages. On the one hand, SRCKF avoids computing the square root of a matrix by directly calculating the square root of the predicted value of error covariance and the estimated value of error covariance. On the other hand, in the SRCKF algorithm, the symmetry and positive qualitative value of the error covariance matrix can always be guaranteed.

During the derivation of the CKF algorithm, it is generally assumed that the statistical characteristics of system noise and measurement noise are known [21]. However, in practice, the statistical characteristics of noise are often unknown and time-varying. The Sage–Husa estimator is often used to estimate the statistical characteristics of noise because of its simplicity and good real-time performance [22]. However, the conventional Sage–Husa estimator is suitable for estimating the statistical properties of constant coefficient noise in linear systems [23]. Based on the conventional Sage–Husa algorithm, an adaptive noise statistical estimator for non-linear systems is derived by using the cubature rule.

For time-varying noise statistics, the real-time updated data play a leading role, while the old data play a small role compared with the new data. Therefore, we should gradually reduce the weight of old data and increase the weight of new data. The exponential weighted attenuation method for fading memory is introduced to estimate time-varying noise. The exponential weighted attenuation method has the characteristic of remembering the past historical data, but the weighted coefficient of the old data is small [24].

The current international standard “ISO/DIS15622 Intelligent transportation systems-adaptive cruise control systems-performance requirements and test procedures” clearly states that adaptive cruise control (ACC) may ignore stationary targets or do not respond to stationary targets. At the same time, for full-speed ACC and autonomous emergency braking (AEB) systems, it is necessary to accurately identify the target-vehicle as a stationary target-vehicle or a moving target-vehicle.

The recognition of the target motion state has the following two functions for the ADAS. On the one hand, it can predict the future trajectory of the target-vehicle; On the other hand, it can determine the degree of danger of the target-vehicle to the ego-vehicle. Therefore, it is essential to study the classification of the target-vehicle motion state. In the literature [25], targets detected by radar are divided into high-altitude targets, stationary targets, moving targets, and road targets, but the basis for target classification is not discussed in detail. Therefore, a method of classifying the motion state of the target-vehicle based on a time window is proposed by analyzing the transfer mechanism of the motion state of the target-vehicle.

The motivation of writing the paper is as follows: (1) For the on-board millimeter-wave radar in the unknown and time-varying noise environment, the accuracy of a high-dimensional non-linear target tracking process is low. The ISRCKF algorithm based on SRCKF is proposed to accurately estimate the unknown and time-varying noise statistics. (2) To accurately predict the future trajectory of the target vehicle and determine the danger degree of the target vehicle to the ego vehicle. We present a classification method for moving objects and stationary objects based on the mechanism of moving state transfer in a time window. The vehicle test results show: (1) The filter accuracy of the ISRCKF algorithm is higher than that of SRCKF and SH-EKF. (2) The classification and recognition results of the target-vehicle’s motion state are consistent with the target-vehicle’s motion state. 

The rest of the paper is organized as follows. Section 2, based on the Cartesian coordinate system of millimeter-wave radar, the target-vehicle motion state model is established; In Section 3, based on the SRCKF, an adaptive square-root cubature Kalman filter (ASRCKF) is derived. In Section 4, based on the analysis of the motion state and transfer principle of the target, a classification method of moving target and stationary target based on the motion state transfer mechanism in a time window is proposed. In Section 5, the algorithm is validated and its results are analyzed by establishing a vehicle test platform. Section 6 presents the conclusions.

## 2. Motion Model of Target-Vehicle

### 2.1. Coordinate System

It can be learned from the dynamics that the description of the same target motion state varies in different reference coordinate systems. Therefore, it is meaningful to clarify the coordinate system that describes the target motion. 

The target measurement information (relative radial distance, azimuth, and relative radial speed) measured by the millimeter-wave radar is obtained from the millimeter-wave radar polar coordinate system. This paper studies the target tracking algorithm based on the millimeter-wave radar Cartesian coordinate system, to verify the performance and accuracy of the proposed algorithm in the target tracking process.

**Figure 1:**
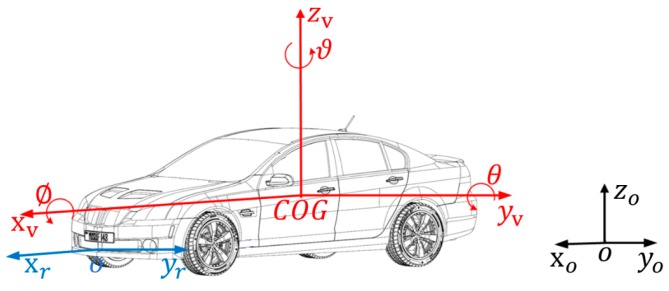
Coordinate system.

The three coordinate systems herein are respectively, the geodetic coordinate system $x_{o}y_{o}z_{o}$, on the horizontal ground, the vehicle motion coordinate system $x_{v}y_{v}z_{v}$ with its origin coinciding with the center of gravity vehicle, the millimeter-wave radar coordinate system $x_{r}oy_{r}$, as shown in the Figure 1. The millimeter-wave radar is fixedly mounted on the front of the vehicle and the radar beam is aligned with the longitudinal axis of the vehicle. Therefore, the millimeter-wave radar coordinate system $x_{r}oy_{r}$ is parallel to the vehicle motion coordinate system $x_{v}y_{v}z_{v}$. The radar $x_{r}$ axis direction is identical with the $x_{v}$ direction in the vehicle motion coordinate system. The radar $y_{r}$ axis direction is identical with the $y_{v}$ direction in the vehicle motion coordinate system.

### 2.2. Equation of Motion

The forward millimeter-wave radar is installed in the middle of the front bumper and fixed to the vehicle body. As the vehicle travels, the millimeter-wave radar detects the target in front of the vehicle, mainly referring to the target-vehicles. These target-vehicles have no vertical motion or small moving speed in the vertical direction, so only the movement of the target-vehicles in the XY plane needs to be considered. Since the target-vehicles motion state has the characteristics of small mobility and low speed, a constant velocity model is established based on the millimeter-wave radar Cartesian coordinate system to describe the motion state of the front target-vehicles.

The model of the constant velocity of the target-vehicle can be obtained from the millimeter-wave radar Cartesian coordinate system:

(1) $$\left\{ \begin{array}{l} x\left(k+1\right)=x\left(k\right)+\dot{x}\left(k\right)*T+\frac{1}{2}w_{x}\left(k\right)*T^{2}\\ \dot{x}\left(k+1\right)=\dot{x}\left(k\right)+w_{x}\left(k\right)*T\;\\ y\left(k+1\right)=y_{\left(k\right)}+\dot{y}\left(k\right)*T+\frac{1}{2}w_{y}\left(k\right)*T^{2}\\ \dot{y}\left(k+1\right)=\dot{y}\left(k\right)+w_{y}\left(k\right)*T \end{array}\right.$$

where (x(k+1),y(k+1)) represents the longitudinal distance and the lateral distance from the forward target-vehicles in the millimeter-wave radar Cartesian coordinate system at k+1, respectively. $\left(\dot{x}\left(k+1\right),\dot{y}\left(k+1\right)\right)$ is the longitudinal velocity and the lateral velocity of the target-vehicles relative to the millimeter-wave radar Cartesian coordinate system movement at k+1. T is the sampling time.

The model of the target motion state is as follows:

(2) X(k+1)=A∗X(k)+B∗w(k)

The target motion state vector $X={\left[x,\dot{x},y,\dot{y}\right]}^{T}$ here is designed to describe the motion state of the target-vehicle in the millimeter-wave radar Cartesian coordinate system; where, ***A*** is the state transition matrix, ***B*** noise-driven matrix, and w(k) the measurement noise at k;

$$A=\left[\begin{array}{c} \begin{array}{ccc} 1 & T & \begin{array}{cc} 0 & 0 \end{array} \end{array}\\ \begin{array}{c} \begin{array}{ccc} 0 & 1 & \begin{array}{cc} 0 & 0 \end{array} \end{array}\\ \begin{array}{ccc} 0 & 0 & \begin{array}{cc} 1 & T \end{array} \end{array}\\ \begin{array}{ccc} 0 & 0 & \begin{array}{cc} 0 & 1 \end{array} \end{array} \end{array} \end{array}\right]\;\;\;\;B=\left[\begin{array}{cc} \begin{array}{c} T^{2}/2\\ \begin{array}{c} T\\ 0\\ 0 \end{array} \end{array} & \begin{array}{c} 0\\ \begin{array}{c} 0\\ T^{2}/2\\ T \end{array} \end{array} \end{array}\right]$$

The target motion state information of millimeter-wave radar in a polar coordinate system is transformed to the front target vehicle in the millimeter-wave radar Cartesian coordinate system by coordinate transformation, the conversion formula is:

(3) $$\{\begin{array}{c} x=R*\cos\theta \\ y=R*\sin\theta \end{array}$$

where (x,y) is the position of the target-vehicle in the millimeter-wave radar Cartesian coordinate system θ is the azimuth.

The measurement equation of the target-vehicle in a millimeter-wave radar Cartesian coordinate system:

(4) $$Z\left(k+1\right)=\{\begin{array}{c} R\left(k+1\right)=\sqrt{x^{2}\left(k+1\right)+y^{2}\left(k+1\right)}+\nu_{1}\left(k+1\right)\\ V\left(k+1\right)=\sqrt{{\dot{x}}^{2}\left(k+1\right)+{\dot{y}}^{2}\left(k+1\right)}+\nu_{2}\left(k+1\right) \end{array}$$

where $v_{i}\left(k\right)$, i=1,2 are the observation noises at k.

Equations (2) and (4) are the state equation and the measurement equation, respectively. As can be seen from the system model, the state equation is linear and the measurement equation is non-linear. Since the measurement equation is non-linear, the target tracking based on the millimeter-wave radar Cartesian coordinate system in a non-linear system.

### 2.3. Parameters of the Millimeter-Wave Radar

There are two main frequency bands for automobile millimeter-wave radar: 77 GHz and 24 GHz. The 24 GHz millimeter-wave radar is usually installed on the side of the vehicle, the detection range is small and mainly used for blind spot detection (BSD), lane change assistance (LCA)**.** The 77 GHz millimeter-wave radar has a large detection range and is usually installed in front of the vehicle. It is mainly used for forward collision warning (FCW) and AEB. In order to verify the accuracy of the algorithm’s estimation of the motion state of the target vehicle ahead, a 77 GHz millimeter-wave radar is used.

Table 1 shows the specific technical specifications of the on-board millimeter-wave radar provided by the supplier. Its main technical parameters such as detection range, measurement accuracy, and resolution are usually provided in its specification. A 77 GHz millimeter-wave radar is used herein provided by a supplier. The radar data rate is 40 ms. There are long-distance radar and medium-distance radar working states, which are subject to the driving speed of the ego-vehicle. When the travel speed of ego-vehicle is greater than 80 km/h and the millimeter-wave radar is in the long-distance radar working mode, it achieves the farthest detection distance of 120 m, and the angle detection range of around 30°. When the travel speed of the ego-vehicle is less than 80 km/h and the millimeter-wave radar is in the medium-distance radar working mode, it reaches the farthest detection distance of 100 m, and the angle detection range of around 50°.

## 3. Adaptive Square-Root Cubature Kalman Filter

This section is based on SRCKF. The Sage–Husa noise statistic estimator is extended from the linear constant noise statistic estimator to the non-linear time-varying noise statistic estimator.

### 3.1. Cubature Rule

CKF is derived from Bayesian filter theory in the Gaussian field. Under the framework of Bayesian filter theory in Gaussian domain, the non-linear filter problem can be summarized as a weighted integral in the form of “*non-linear* × *Gaussian density*”. that is:

(5) $$I\left(f\right)=\int_{Ω}f\left(x\right)\omega \left(x\right)dx$$

where f(x) is a non- linear function, $Ω\subseteq R^{n}$ is the region of integration, $\omega \left(x\right)=\exp\left(-x^{T}x\right)$ is a Gaussian density and satisfies the non-negativity condition in the entire region.

CKF uses the third-degree spherical-radial rule to calculate the non-linear filter problem [17]. Based on the third-degree spherical-radial rule, a set of 2n equal weight sampling points is used to approximate the integral value. that is:

(6) $$I\left(f\right)\approx \overset{2n}{\underset{i=1}{\sum }}\omega_{i}f\left(\xi_{i}\right)$$

where $\xi_{i}=\sqrt{n}{\left[1\right]}_{i}$ is the cubature point, $\omega_{i}=\frac{1}{2n}$ is the corresponding weight, i=1, …,2n, ${\left[1\right]}_{i}$ is the no.i element of the cubature points set:

(7) $${\left[1\right]}_{i}=\{\left[\begin{array}{c} 1\\ \begin{array}{c} 0\\ \begin{array}{c} \vdots \\ 0 \end{array} \end{array} \end{array}\right],\left[\begin{array}{c} 0\\ \begin{array}{c} 1\\ \begin{array}{c} \vdots \\ 0 \end{array} \end{array} \end{array}\right]\ldots \left[\begin{array}{c} 0\\ \begin{array}{c} 0\\ \begin{array}{c} \vdots \\ 1 \end{array} \end{array} \end{array}\right],\left[\begin{array}{c} -1\\ \begin{array}{c} 0\\ \begin{array}{c} \vdots \\ 0 \end{array} \end{array} \end{array}\right],\left[\begin{array}{c} 0\\ \begin{array}{c} -1\\ \begin{array}{c} \vdots \\ 0 \end{array} \end{array} \end{array}\right]\ldots \left[\begin{array}{c} 0\\ \begin{array}{c} 0\\ \begin{array}{c} \vdots \\ -1 \end{array} \end{array} \end{array}\right]\}$$

### 3.2. Square-Root Cubarure Kalman Filter

In CKF, the error covariance matrix needs to satisfy two conditions: (1) symmetry (2) positive qualitative. It is important to ensure these two points in the algorithm iteration process to improve the robustness of the filter. The robustness of algorithm is defined as free of failure that the parameters in the algorithm violate the constraints, which makes the algorithm unable to continue running.

In order to ensure the symmetry and positive qualitative of the error covariance matrix, Arasaratnam proposed the SRCKF algorithm based on the CKF algorithm [17]. The SRCKF algorithm process is as follows:

(1) Initialization

${\hat{x}}_{k}$ is the state variable, $P_{k}$ is the error covariance matrix, $Q_{k}$ is the process noise, $R_{k}$ is the measurement noise.

(2) Evaluate the cubature points:

(8) $$X_{j,k}=S_{k}\xi_{j}+{\hat{x}}_{k};\;j=1,2,\ldots ,2n$$

where $\xi_{j}$ is the cubature points set, as shown below:

$$\xi_{j}=\{\begin{array}{cc} \sqrt{n}{\left[1\right]}_{i} & i=1,2,\ldots ,n\\ -\sqrt{n}{\left[1\right]}_{i} & \;i=n+1,n+2,\ldots ,2n \end{array}$$

where [1] is the unit matrix.

(3) Spread cubature points and calculate the state prediction:

(9) $$X_{j,k+1}^{*}=f\left(X_{j,k},u_{k}\right)$$

(10) $${\bar{x}}_{k+1}=\frac{1}{2n}*\overset{2n}{\underset{j=1}{\sum }}X_{j,k+1}^{*}$$

(4) Estimate the square-root factor of the predicted error covariance

(11) $${\bar{S}}_{k+1}=Tria\left(\left[\chi_{k+1}^{*}S_{Q,k+1}\right]\right)$$

where Tria() represents a triangular operation. B=Tria(A) means: B is a matrix of $A^{T}$ upper triangular matrix obtained QR decomposition. 

$\chi_{k+1}^{*}$ and $S_{Q,k+1}$ are as follows:

$$\begin{array}{c} \chi_{k+1}^{*}=1/\left(\sqrt{2n}\right)\left[X_{1,k+1}^{*}-{\bar{x}}_{k+1},\;X_{2,k+1}^{*}-{\bar{x}}_{k+1},\cdots ,X_{2n,k+1}^{*}-{\bar{x}}_{k+1}\right]\\ S_{Q,k+1}=\mathrm{chol}\left(Q_{k+1}\right) \end{array}$$

where chol() is Cholesky decomposition.

(5) Recalculate the cubature points

(12) $$X_{j,k+1}={\bar{S}}_{k+1}\xi_{j}+{\bar{x}}_{k+1}$$

(6) Spread cubature points

(13) $$Z_{j,k+1}=h\left(X_{j,k+1},u_{k+1}\right)$$

(7) Observation Prediction:

(14) $${\bar{z}}_{k+1}=\frac{1}{2n}\overset{2n}{\underset{j=1}{\sum }}Z_{j,k+1}$$

(8) Estimate the square-root of the innovation covariance matrix:

(15) $$S_{ZZ,k+1}=Tria\left(\left[\gamma_{k+1}\;S_{R,k+1}\right]\right)$$

where $\gamma_{k+1}$ and $S_{R}$ are as follows:

$$\begin{array}{c} \gamma_{k+1}=1/\left(\sqrt{2n}\right)\left[Z_{1,k+1}-{\bar{z}}_{k+1},\;Z_{2,k+1}-{\bar{z}}_{k+1},\cdots ,Z_{2n,k+1}-{\bar{z}}_{k+1}\right]\\ S_{R,k+1}=chol\left(R_{k+1}\right) \end{array}$$

(9) Estimate the cross-covariance matrix

(16) $$P_{XZ,k+1}=\chi_{k+1}\gamma_{k+1}^{T}$$

where $\chi_{k+1}$ is as follows:

$$\chi_{k+1}=1/\left(\sqrt{2n}\right)\left[X_{1,k+1}-{\bar{x}}_{k+1},X_{2,k+1}-{\bar{x}}_{k+1},\cdots ,\;X_{2n,k+1}-{\bar{x}}_{k+1}\right]$$

(10) Estimate the Kalman gain

(17) $$K_{k+1}=(P_{XZ,k+1}/S_{zz,k+1}^{T})/S_{zz,k+1}$$

(11) Estimate the updated state

(18) $${\hat{x}}_{k+1}={\bar{x}}_{k+1}+K_{k+1}\left(z_{k+1}-{\bar{z}}_{k+1}\right)$$

(12) Estimate the square-root factor of the corresponding error covariance

(19) $$S_{k+1}=Tria\left(\left[\chi_{k+1}-K_{k+1}\gamma_{k+1}K_{k+1}S_{R}\right]\right)$$

### 3.3. Improved Square-Root Cubature Kalman Filter

The on-board millimeter-wave radar is driving in the roads, and the statistical parameters of measurement noise are often unknown and time-varying. If the values of measurement noise and process noise are not consistent with the actual noise statistics, the filter will diverge. Therefore, constructing the ASRCKF for online estimation of unknown noise statistics is of great significance for improving the accuracy of filters. Sage-Husa is often used to estimate the statistical characteristics of noise online because of its simplicity and good real-time performance. However, the conventional Sage–Husa noise statistical estimator is suitable for the constant coefficient noise statistical characteristic estimation of linear systems. In the literature [26], the Sage–Husa noise statistic estimator in linear system is extended to the non-linear Sage–Husa noise statistic estimator. Therefore, based on the literature [26], we combine the Sage–Husa noise statistical estimator and cubature rules to derive a time-varying noise statistical estimator suitable for nonlinear systems.

The noise statistical estimator based on Sage–Husa in a non-linear system is:

Recursive formula for the process noise means:

(20) $${\hat{q}}_{k+1}=\frac{1}{k+1}[k*q_{k}+{\hat{x}}_{k+1}-\frac{1}{2n}\overset{2n}{\underset{i=1}{\sum }}f_{k}\left(X_{i,k|k}\right)]$$

Recursive formula for the measurement noise means:

(21) $${\hat{r}}_{k+1}=\frac{1}{k+1}[k*{\hat{r}}_{k}+z_{k+1}-\frac{1}{2n}\overset{2n}{\underset{i=1}{\sum }}h\left(X_{i,k+1|k}\right)]$$

The process noise variance is written in the form of a recursive estimation formula as:

(22) $$\begin{array}{c} {\hat{Q}}_{k+1}=\frac{1}{k+1}[k*{\hat{Q}}_{k}+K_{k+1}v_{k+1}v_{k+1}^{T}K_{k+1}^{T}+P_{k+1}\\ \;\;\;\;\;\;\;\;-(\frac{1}{2n}\overset{2n}{\underset{i=1}{\sum }}X_{i,k+1|k}^{*}X_{i,k+1|k}^{*T}-{\hat{x}}_{k+1|k}{\hat{x}}_{k+1|k}^{T})] \end{array}$$

The measurement noise variance is written in the form of a recursive estimation formula as:

(23) $${\hat{R}}_{k+1}=\frac{1}{k+1}[k*{\hat{R}}_{k}+v_{k+1}v_{k+1}^{T}-(\frac{1}{2n}\overset{2n}{\underset{i=1}{\sum }}Z_{i,k+1|k}Z_{i,k+1|k}^{T}-{\hat{z}}_{k+1|k}{\hat{z}}_{k+1|k}^{T})]$$

Including: $\;\hat{q}$, $\hat{r}$, $\hat{Q}$, $\hat{R}$ are the maximum a posterior of q, r, Q, R respectively.

As can be seen from (20), (21), (22), (23), the weight coefficient of each factor in k+1 moments, every factor of the weighted coefficient of $\hat{q}$, $\hat{r}$, $\hat{Q}$, $\hat{R}$ is 1/(k+1). For time-varying noise, the role of new data should be increased, while the role of old data should be gradually forgotten. Therefore, a different weighting factor should be multiplied for each factor in the system noise. That is: the weighting coefficient of new data should be greater than that of the old data. On the basis of the constant noise statistic estimator, the time-varying noise statistic estimator suitable for SRCKF is deduced by using the method of fading memory index weighting.

Weighted coefficient: $\lambda_{i}=\lambda_{i-1}*b$, 0<b<1, $\sum_{i=1}^{k+1}\lambda_{i}=1$.

Therefore, the weighted index of fading memory is:

(24) $$\{\begin{array}{c} \lambda_{i}=d_{k}b^{i-1}\\ d_{k}=\frac{1-b}{1-b^{k}} \end{array}$$

where b is the forgetting factor, and its value range is usually between 0.95 and 0.99. A time-varying noise statistical estimator is obtained by replacing $\lambda_{k+1-i}$ with the weight factor of 1/(k+1) in the constant noise statistical estimator:

Recursive formula for the process noise means:

(25) $${\hat{q}}_{k+1}=\left(1-d_{k+1}\right){\hat{q}}_{k}+d_{k+1}[{\hat{x}}_{k+1|k+1}-\frac{1}{2n}\overset{2n}{\underset{i=1}{\sum }}f_{k}\left(X_{i,k|k}\right)]$$

Recursive formula for the measurement noise means:

(26) $${\hat{r}}_{k+1}=\left(1-d_{k+1}\right){\hat{r}}_{k}+d_{k+1}[z_{k+1}-\frac{1}{2n}\overset{2n}{\underset{i=1}{\sum }}h_{k+1}\left(X_{i,k+1|k}\right)]$$

The process noise variance is written in the form of a recursive estimation formula as:

(27) $$\begin{array}{c} {\hat{Q}}_{k+1}=\left(1-d_{k+1}\right){\hat{Q}}_{k}+d_{k+1}[K_{k+1}\varepsilon_{k+1}\varepsilon_{k+1}^{T}K_{k+1}^{T}+P_{k+1}\\ \;\;\;\;\;-(\frac{1}{2n}\overset{2n}{\underset{i=1}{\sum }}X_{i,k+1|k}^{*}X_{i,k+1|k}^{*T}-{\hat{x}}_{k+1|k}{\hat{x}}_{k+1|k}^{T})] \end{array}$$

where

$$\varepsilon_{k}=z_{k}-{\hat{z}}_{k|k-1}$$

The measurement noise variance is written in the form of a recursive estimation formula as:

(28) $${\hat{R}}_{k+1}=\left(1-d_{k+1}\right){\hat{R}}_{k}+d_{k+1}[\varepsilon_{k+1}\varepsilon_{k+1}^{T}-(\frac{1}{2n}\overset{2n}{\underset{i=1}{\sum }}Z_{i,k+1|k}Z_{i,k+1|k}^{T}-{\hat{z}}_{k+1|k}{\hat{z}}_{k+1|k}^{T})]$$

## 4. Classification of Target-Vehicle Motion State

Due to the influence of the ego-vehicle speed sensor and millimeter-wave radar measurement error, the direct use of the current moment of the ego vehicle and target vehicle motion relationship to identify the target-vehicle motion state, will lead to the vibration and even inaccurate motion state classification results. A method of classifying the motion state of the target vehicle based on time window is proposed by analyzing the transfer mechanism of the motion state of the target-vehicle. According to the absolute velocity of the target vehicle, the motion state of the target vehicle is divided into stationary target vehicle, moving target vehicle, oncoming target vehicle, start-stop target vehicle, and unclassified target vehicle. 

(1) Unclassified target vehicle: the motion state of the target vehicle obtained at the initial moment of radar is the unclassified target vehicle;

(2) Stationary target vehicle: the target vehicle whose absolute speed stays near zero for a long time;

(3) Moving the target vehicle in the same direction: the movement direction of the target vehicle is the same as that of the ego vehicle;

(4) Oncoming target vehicle: the movement direction of the target vehicle is opposite to that of the ego vehicle;

(5) Start-stop target vehicle: the speed of the moving target vehicle (or the oncoming target vehicle) is reduced to near zero.

Since the velocity measured by the on-board millimeter-wave radar is the relative motion velocity of the target vehicle relative to the ego vehicle. Therefore, the absolute velocity of the target vehicle relative to the geodetic coordinate system can be deduced:

(29) $$V_{1}=V_{r}+V_{v}$$

where:

$V_{1}$: The absolute velocity of the target vehicle;
$V_{r}$: The relative velocity of the target vehicle;
$V_{v}$: The speed of the ego vehicle.

**Figure 2:**
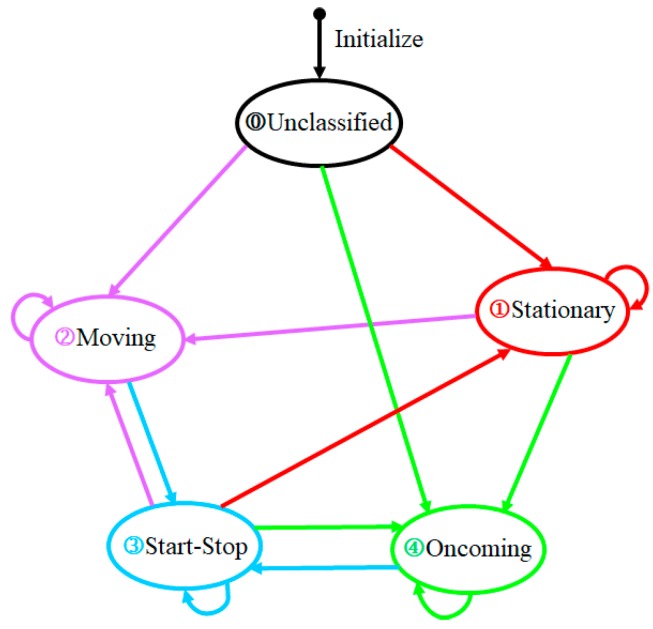
The flow chart of movement state transfer of the target vehicle.

Figure 2 shows the flow chart of movement state transfer of the target vehicle.

According to the absolute speed of the target vehicle, the determination of the target motion state is mainly influenced by the following two factors: first, the measurement error of the ego vehicle speed sensor; Second, millimeter-wave radar speed error. Due to the above two measurement errors, the stationary target may also return a non-zero velocity value. Therefore, it is essential to determine the appropriate threshold value to judge the target motion state. The influence of velocity sensor measurement error and millimeter-wave radar measurement error is considered. In this paper, the reference ranges of the velocity threshold are [0.5~1m/s] and [−1~−0.5m/s]. Because the fluctuation range of the ego-vehicle velocity is 0.3m/s. Therefore, the threshold value of the velocity of the stationary target-vehicle is set to ±0.5m/s. The moving state transition rules of the target vehicle are as follows:

(1) The motion state of the target vehicle obtained during the initial operation of the radar is unclassified;

(2) The absolute speed of the target vehicle is between [−0.5~0.5m/s] for N consecutive cycles. The target motion state transition can be divided into the following four conditions:

① Switch from unclassified to stationary;

② Keep stationary;

③ Switch from oncoming to start-stop;

④ Switch from moving to starting-stopping;

(3) When the absolute speed of the target-vehicle is greater than 0.5m/s for N consecutive cycles, the target motion state transition has the following four conditions:

① Switches from unclassified to moving;

② Keep moving;

③ Switch from stationary to moving;

④ Switch from start-stop to moving;

(4) When the absolute speed of the target-vehicle is less than –0.5 m/s for N consecutive cycles, the target motion state transition has the following four conditions:

① Switch from unclassified to oncoming;

② Keep oncoming;

③ Switch from stationary to oncoming;

④ Switch from start-stop to oncoming;

(5) By recording the time T when the target vehicle is recognized as the start-stop motion state, the transition relationship between the start-stop motion state and the stationary state is identified.

① If T is greater than or equal to M, the target vehicle is switched from start-stop motion state to the stationary state.

② If T is less than M, the target vehicle keeps start-stop motion state.

As the length of time window N is longer, the delay of target-vehicle motion state recognition is more serious. The value of M has a significant influence on decision-making and control of the vehicle. In this paper, N=3, M=2s.

Because the target vehicle has inertia, there is no sudden change in the speed of the target-vehicle. In the process of state transfer between moving target vehicles in the same direction and moving target vehicles in the opposite direction, it is necessary to go through the start-stop motion state.

## 5. Experiment and Discussion

### 5.1. Construction of the Experimental Platform

**Figure 3:**
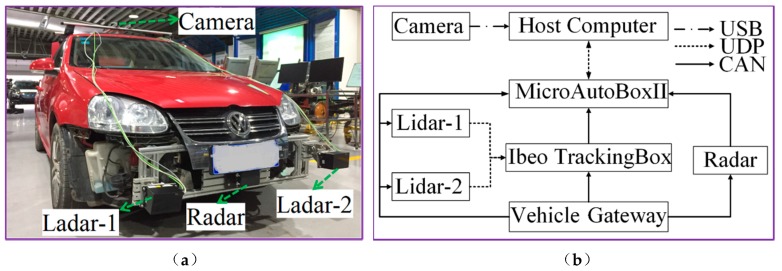
Experimental vehicle. (**a**) Test platform equipment, (**b**) experimental platform communication.

As shown in the Figure 3, to check the accuracy and reliability of the proposed algorithm, the Sagitar vehicle is applied herein in the test. Figure 3 (a) is the Sagitar test vehicle platform; (b) is the experimental platform communication.

The ego-vehicle is equipped with a 77 GHz millimeter-wave radar as shown in (a) and (b) of Figure 3. The 77 GHz millimeter-wave radar is installed directly above the license plate on the front of the ego-vehicle. The 77 GHz millimeter-wave radar is provided with two-way controller area network (CAN). One CAN is connected to the vehicle gateway. The other is connected to MicroAutoBoxII through CAN. The AR023Z-1080p camera is installed in the bracket above the ego-vehicle to synchronously record the scene of the vehicle. 

**Figure 4:**
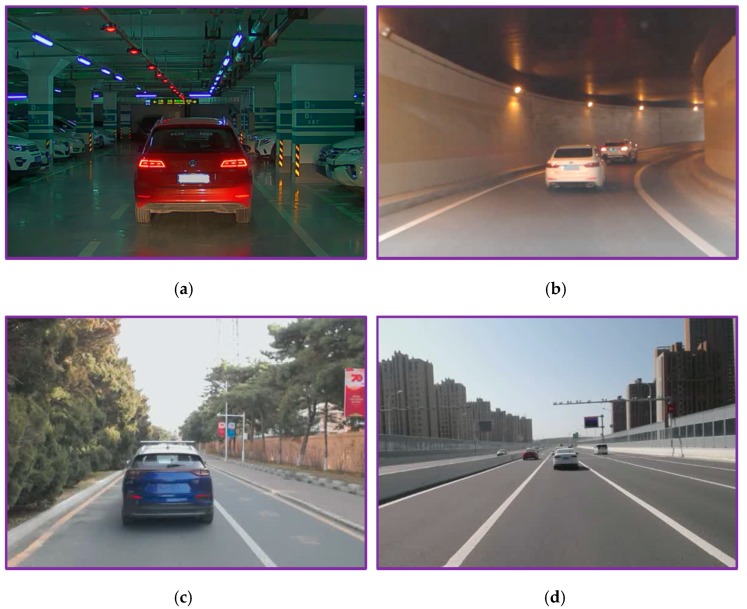
Experimental environment. (**a**) underground parking, (**b**) tunnel, (**c**) campus, (**d**) expressways.

We use the light detection and ranging (lidar) produced by Ibeo to detect the motion state information of the target vehicle and take it as the ground truth, which is used to verify the performance of the target tracking algorithm of the millimeter-wave radar. In order to make the sensors smarter, Ibeo will provide point cloud processing algorithm software for the lidar. At present, the algorithm provided by Ibeo supports target recognition and tracking. The motion state information of the target vehicle includes longitudinal distance and longitudinal speed. As shown in Figure 4a, the ego-vehicle is equipped with two lidars, which are lidar-1 and lidar-2. The Ibeo TrackingBox produced by Ibeo is responsible for data fusion of the two lidars. Table 2 shows the specific technical specifications of the four scan levels lidar provided by Ibeo.

The ego-vehicle is equipped with MicroAutoBoxII and connected to the vehicle gateway port via the CAN to obtain the longitudinal velocity and steering wheel angle information of the vehicle measured by the electronic stability controller (ESC). MicroAutoBoxII is connected to the Ibeo TrackingBox through CAN and obtains the target vehicle movement status after data fusion. 

The motion state estimation algorithm of the target vehicle is written in the environment of MatlabR2018a/Simulink in the host computer. The automatic code generation software provided by the dSPACE company is used to download the code to the MicroAutoBoxII1401/ 1505/1507 rapid prototyping controller through user datagram protocol (UDP) to run.

In order to exclude the randomness of the experiments, we conducted multiple sets of experiments. As shown in Figure 4, four test environments are selected, and three groups of experiments are carried out for each test environment.

### 5.2. Analysis of Experiment Results

**Figure 5:**
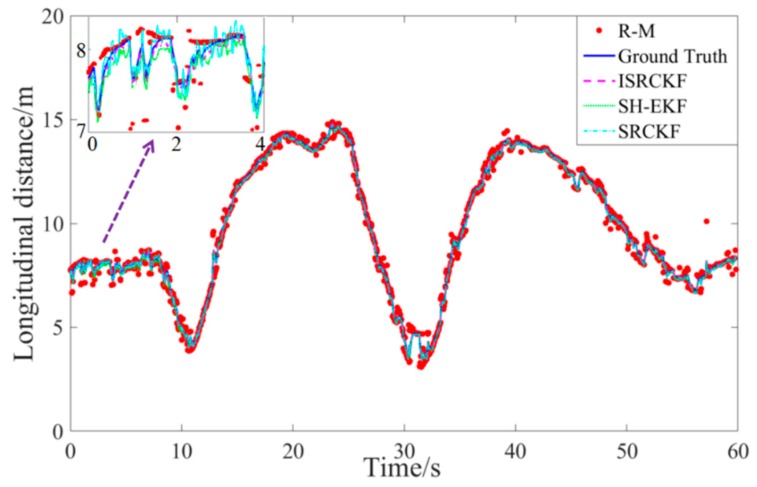
Target-vehicle longitudinal distance time history curve.

**Figure 6:**
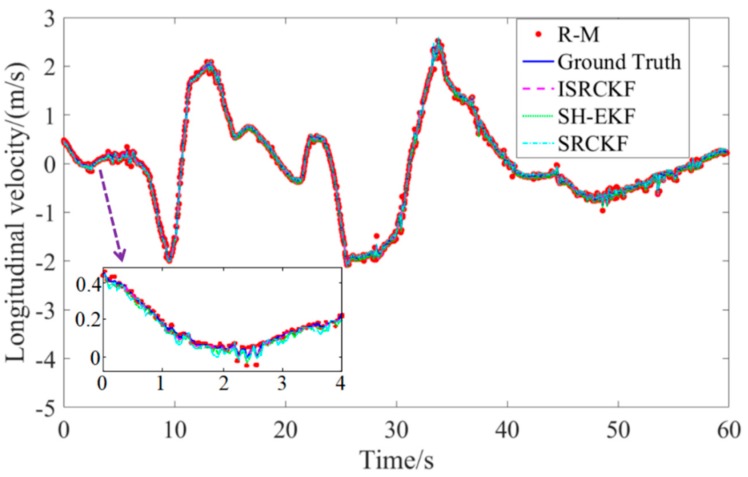
Target-vehicle longitudinal velocity time history curve.

Since the test results for the 12 groups of experiments are similar, one set of test data is selected for discussion and analysis. Figure 5 to Figure 6 show the experimental results of the effect of verifying the ISRCKF target tracking algorithm.

The measurement data of the target vehicle are indicated by the red circle, without the ISRCKF algorithm optimization and containing noise (R-M). The solid blue line represents the ground truth of the motion state of the target-vehicle measured by lidar. The measurement data of the target vehicle are indicated by the magenta dashed line, with the ISRCKF algorithm optimization. The measurement data of the target vehicle are indicated by the green solid line, with the SH-EKF algorithm optimization. The measurement data of the target vehicle are indicated by the cyan dot-dash line, with the SRCKF algorithm optimization. 

**Figure 7:**
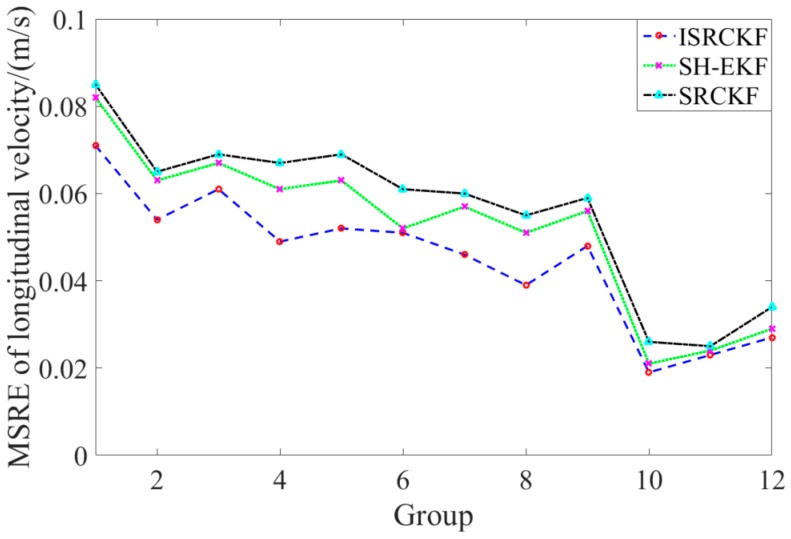
MSRE curves of longitudinal velocity for various filters.

**Figure 8:**
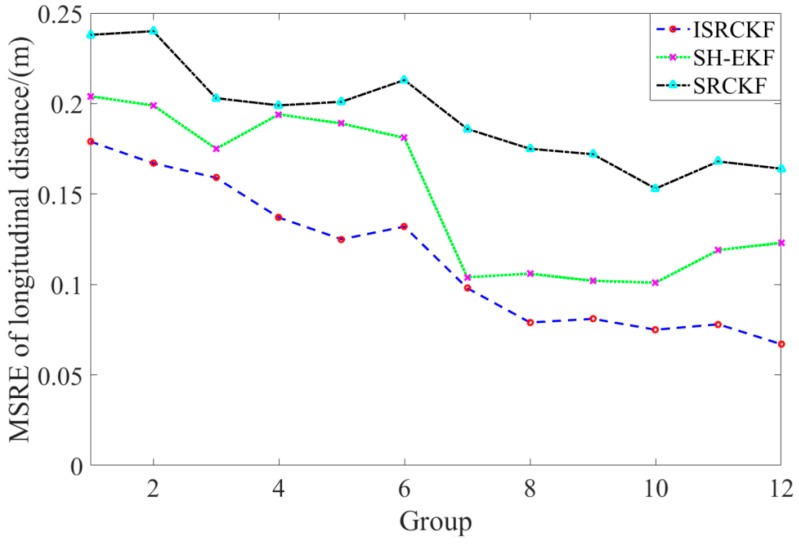
MSRE curves of longitudinal distance for various filters.

Figure 5 and Figure 6 show the time history curves of the longitudinal distance and longitudinal velocity of the target-vehicle relative to the ego-vehicle. It can be concluded from the figures that the data fluctuation of the ISRCKF algorithm during tracking the target is small, and the target tracking performance of the ISRCKF algorithm is significantly better than the SH-EKF and SRCKF algorithms. Through references [27,28,29], it is found that the root mean square error (RMSE) can be used to quantitatively analyze the filtering accuracy. In order to quantitatively analyze the filtering accuracy of the ISRCKF, SH-EKF, and SRCKF algorithms, we counted the RMSE of the 12 groups of experiments. and the results are shown in Figure 7 and Figure 8. The test environment of the statistical value for RMSE of groups 1, 2, and 3 is the underground parking. The test environment of the statistical value for RMSE of groups 4, 5, and 6 is the tunnel. The test environment of the statistical value for RMSE of groups 7, 8, and 9 is the campus. The test environment for the statistical value for RMSE of groups 10, 11, and 12 is the expressway. 

It can be concluded from Figure 7 and Figure 8 that in the driving environment where the system noise is unknown and time-variant, the RMSE of the longitudinal speed and longitudinal distance of target tracking using SRCKF and SH-EKF algorithm is larger than that of ISRCKF algorithm. Compared with SH-EKF and SRCKF algorithms, the maximum increase in filtering accuracy of longitudinal distance using the ISRCKF algorithm is 45.53% and 59.15%, respectively, and the maximum increase in filtering accuracy of longitudinal speed using the ISRCKF algorithm is 23.53% and 29.09%, respectively. Through the above analysis, it can be concluded that using ISRCKF algorithm to track can effectively suppress the divergence of target tracking, thereby reducing the tracking error and improving the tracking accuracy.

**Figure 9:**
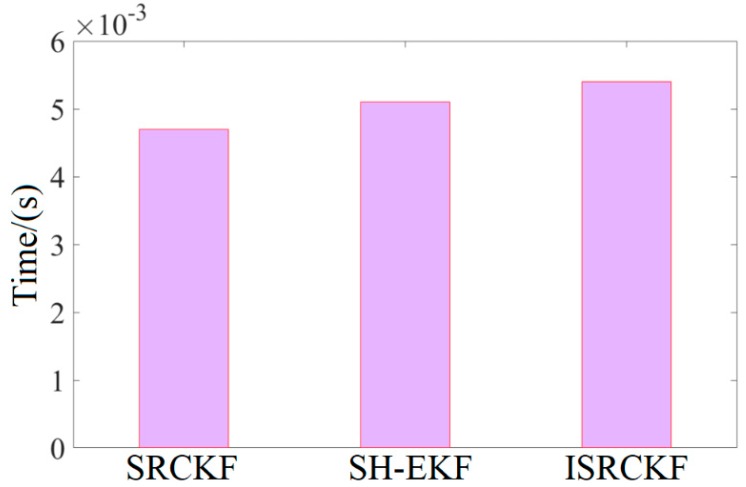
Time mean of the algorithm running once.

As shown in Figure 9, the time consumption of different algorithms is measured by the mean time of the algorithm running once in the MicroAutoBoxII.

From Figure 9, it can be concluded that the target tracking algorithm proposed herein takes 0.0053 s on average, slightly up that of SH-EKF and SRCKF. SH-EKF and SRCKF take 0.0051 s and 0.0047 s on average, respectively. 

The vehicle test results show that the ISRCKF algorithm has the highest accuracy when the time consumption is not increased much compared with SH-EKF and SRCKF.

In order to ensure the safety and rigorous of the experiment, we conducted 10 groups of experiments on campus. During the test, the driving process of the target vehicle was divided into the following stages: parking, accelerated reverse, braking to stop, starting acceleration, and decelerating to stop. Since the test results of the 10 groups are similar, we choose one of the data for analysis.

**Figure 10:**
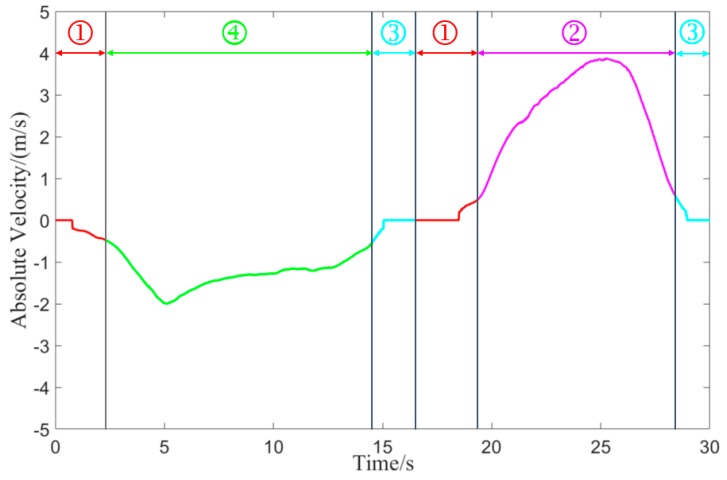
Target-vehicle absolute velocity time history curve.

Figure 10 is the time history curve of the target-vehicle’s absolute velocity. The meanings of ①, ②, ③, and ④ in Figure 10 represent the stationary, moving, start-stop, oncoming movement states in Figure 2, respectively. We have used the same colors to indicate the same motion state in Figure 10 and Figure 2.

**Figure 11:**
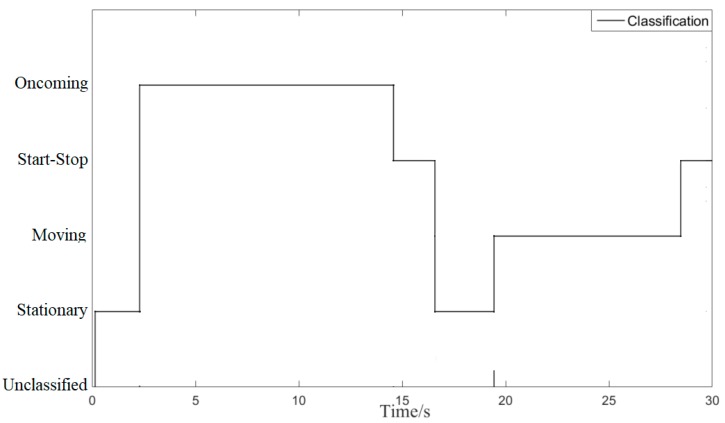
The movement state transfer of the target-vehicle.

Figure 11 is the transition process of the target vehicle’s motion state. The initial motion state of the target vehicle is the unclassified motion state. As the reversing speed of the target vehicle increases at 2.3 s, the motion state of the target-vehicle changes from the stationary motion state to the oncoming motion state. As the reversing speed of the target vehicle decreases, the motion state of the target vehicle changes from the oncoming motion state to the start-stop motion state (at 14.5 s). As the time T of the start-stop motion state increases, the status of the target vehicle changes from the start-stop motion state to the stationary state at the time of 16.5 s. As the forward speed of the target vehicle increases, the motion state of the target vehicle changes from the stationary state to the same motion state (at 19.4 s). As the forward speed of the target vehicle decreases, the motion state of the target vehicle changes from the same motion state to the start-stop motion state at 28.4 s.

In the two sports periods of 14.5 s~16.5 s and 28.4 s~30 s, the classification method proposed classifies target vehicles as start-stop targets. The classification method shows that it has a certain memory effect on the motion state of the target vehicle. The test results show that the classification and recognition results are consistent with the motion state of the target vehicle.

## 6. Conclusions

In the process of on-board millimeter-wave radar target tracking, the characteristics of unknown time-varying noise cannot be accurately counted, which will cause the filter accuracy to decline or even diverge. In response to this question, based on the SRCKF, the Sage–Husa noise statistic estimator and the fading memory exponential weighting method are combined to derive a time-varying noise statistic estimator for non-linear systems. ISRCKF can effectively overcome the problem of low accuracy and even divergence of SRCKF filtering-varying noise. When the motion relation between the ego vehicle and the target vehicle in the current is used to identify the motion state of the target vehicle, there exists the problem that the classification result of motion state is vibration or even inaccurate. A method of classifying the motion state of the target vehicle based on the time window is proposed by analyzing the transfer mechanism of the motion state of the target vehicle. According to the absolute velocity of the target vehicle, the motion state of the target vehicle is divided into stationary target vehicle, moving target vehicle, oncoming target vehicle, start-stop target vehicle, and unclassified target vehicle. Because the target vehicle has inertia, there is no sudden change in the speed of the target vehicle. In this classification method, the target vehicle needs to go through the start-stop motion state during the state transfer between the same motion and the reverse motion. Since the starting and stopping motion state of the target vehicle is different from the stationary motion state of the target vehicle, this method reflects that the target vehicle has a certain memory effect on its motion state. The results of the vehicle test show that: (1) the accuracy of the ISRCKF algorithm is significantly improved; (2) the classification and recognition results of the target vehicle motion state are consistent with the target vehicle motion state.

## Figures and Tables

**Table 1:** Main technical parameters of the millimeter-wave radar.

Parameter	Long-Distance Mode	Medium-Distance Mode
Ranging	1–120/(m)	1–100/(m)
Ranging resolution	0.6(m)	0.2(m)
Angle	±30/(^o^)	±50/(^o^)
Speed resolution	0.75/(km/h)	0.6/(km/h)
Speed	–50–55/(m/s)	–50–55/(m/s)

Remark: ① The horizontal angle range is negative when the target is on the left side of the radar and positive when on the right side. ② The relative speed is positive when the target is far from the radar, and negative when close to the radar.

**Table 2:** Main technical parameters of the lidar.

Parameter	Value/(Units)
Ranging	200(m)
Ranging resolution	0.04(m)
Fov(H*V)	110*3.2/(^o^)
Vertical angle resolution	0.8/(^o^)
update rate	25/(Hz)
